# Monolayer MoS_2_ With Ultrahigh Piezoelectricity: From ALD Dose Control to Device Performance

**DOI:** 10.1002/advs.77323

**Published:** 2026-08-21

**Authors:** Yun Li, Ming Rui Joel Tan, Subhasis Das, Yafei Du, Wenjie Lai, Jiamin Amanda Ong, Yongli He, Yiming Zou, Anatoli Kurkin, Peiwen Huang, Jiajun Liu, Xingyu Chen, Alfred Iing Yoong Tok, Soo Jay Phee, Lioz Etgar, Shlomo Magdassi, Pooi See Lee

**Affiliations:** ^1^ School of Materials Science and Engineering Nanyang Technological University Singapore Singapore; ^2^ Singapore‐HUJ Alliance for Research and Enterprise, The Smart Grippers for Soft Robotics (SGSR) Programme Campus for Research Excellence and Technological Enterprise (CREATE) Singapore Singapore; ^3^ Institute of Molecular and Cell Biology (IMCB) Agency for Science, Technology and Research (A∗STAR) Singapore Singapore; ^4^ Department of Chemistry National University of Singapore Singapore Singapore; ^5^ School of Mechanical and Aerospace Engineering Nanyang Technological University Singapore Singapore; ^6^ Institute of Chemistry The Center for Nanoscience and Nanotechnology Casali Center for Applied Chemistry The Hebrew University of Jerusalem Jerusalem Israel

**Keywords:** atomic layer deposition (ALD), MoS_2_, piezoelectricity, sensors, wearable electronics

## Abstract

A critical challenge in the controllable atomic layer deposition (ALD) synthesis of functional two‐dimensional materials is understanding how early‐stage ultrathin film states govern the final material and device characteristics. Herein, using ALD of MoO_x_ followed by sulfurization, we show that the precursor dosages effectively control the quality of the as‐deposited oxide film and thereby govern the subsequent oxide‐to‐monolayer conversion pathway of MoS_2_. By tuning the ALD dose, the film growth evolves from isolated flakes to porous monolayers and ultimately to continuous monolayers. Comparative structural, chemical, and electronic analyses reveal that the optimized continuous monolayer exhibits higher continuity, lower defect density, improved stoichiometry, and reduced free‐charge screening. As a result, the MoS_2_ monolayer delivers an effective piezoelectric coefficient $d_{11}^{\mathrm{eff}}$ of 4.3 pm V^−1^, while the linear sensitivity of 326.9 mV and 141.38 pA per 1% lateral strain is highest among binary transition metal dichalcogenides. The optimized films also enable proof‐of‐usability demonstrations in wearable sensing and closed‐loop slippage feedback control. These results establish an oxide‐state‐controlled growth framework for engineering continuous low‐screening piezoelectric monolayers and provide a practical route toward high‐performance flexible electromechanical devices.

## Graphical abstract

This study demonstrates that controlling precursor dosages in atomic layer deposition (ALD) serves to regulate ultrathin oxide quality, which in turn determines sulfurization behavior and MoS_2_ monolayer continuity. The resulting continuous, low‐defect monolayers enable improved piezoelectric performance and support a flexible slippage sensor, linking growth control with functional device output.

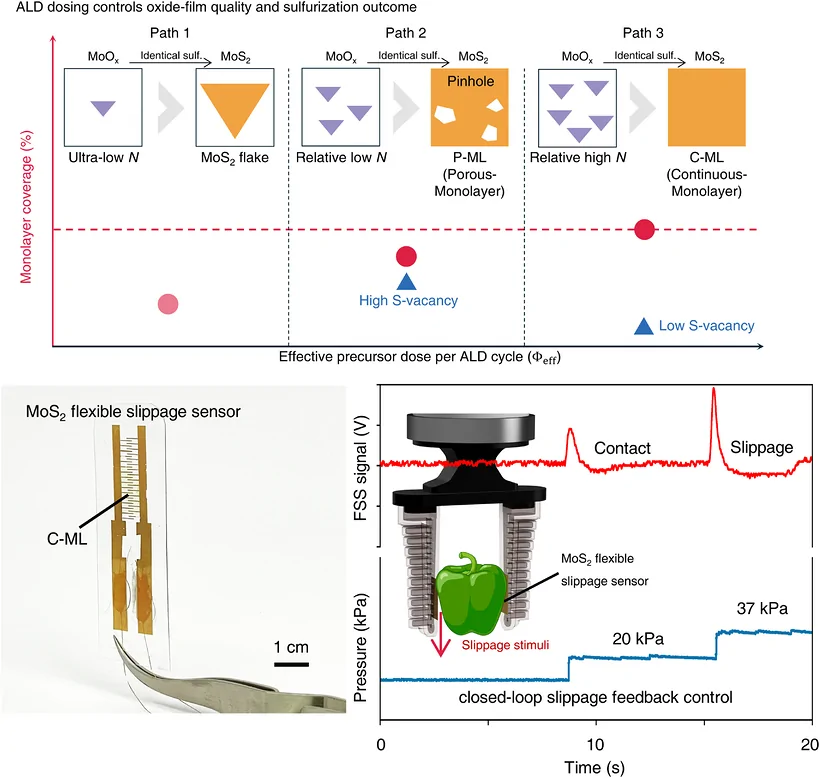

## 1 Introduction

Two‐dimensional transition metal dichalcogenides (2D TMDs) have garnered significant interest due to their unique electronic, optical, and mechanical properties derived from their atomic thickness and tunable bandgap [1, 2, 3, 4, 5]. Among these properties, the intrinsic piezoelectricity in 2D TMDs, arising from their non‐centrosymmetric crystal structures [6, 7, 8], enables highly sensitive and flexible electromechanical responses at the nanoscale. This symmetry‐sensitive behavior makes monolayer control particularly important for realizing robust piezoelectric functionality in practical films. Moreover, the piezoelectric performance of 2D TMD materials is highly sensitive to their film quality, including defect density and film uniformity [9].

However, the synthesis of high‐quality piezoelectric TMD monolayers remains challenging because multiple growth‐related factors, including adsorption, nucleation, and surface diffusion, jointly determine the film continuity, thickness uniformity, and defects formation [10, 11, 12, 13, 14]. At ultrathin film thickness, nucleation delay, random island formation, incomplete coalescence, and defect generation can readily produce porous or nonuniform films, making continuous monolayers difficult to obtain through empirical process optimization alone [15, 16]. To address these challenges, a two‐step procedure involving the deposition of oxides followed by sulfurization, selenization, or tellurization has been developed for the growth of ultrathin TMD films [9, 17]. This two‐step process allows decoupled control of the thickness and crystallinity of the deposited films [18, 19].

Despite these advances, an important mechanistic gap remains in the two‐step growth of ultrathin TMD monolayers. Existing studies have largely focused on sulfurization parameters, such as sulfurization temperature, precursor supply, film thickness, and nucleation behavior, to improve the final chalcogenized films [18, 20, 21, 22]. However, it remains unclear whether the pre‐sulfurization ultrathin oxide‐film itself can act as a variable that determines the subsequent sulfurization pathway and the final monolayer formation. This question is particularly important for piezoelectric monolayers, of which the electromechanical response is highly sensitive to defect‐induced charge screening. In parallel, studies on defect‐screening‐piezoelectricity relationships have mainly emphasized defect passivation or post‐treatment strategies after defect formation [10, 23, 24, 25, 26]. As a result, whether a favorable low‐defect, low‐screening piezoelectric monolayer state can be established during oxide growth and sulfurization, rather than repaired afterward, is still insufficiently understood.

Herein, we use a two‐step route consisting of atomic layer deposition (ALD) of MoO_x_ followed by sulfurization to evaluate how the pre‐sulfurization oxide‐film influences the final monolayer outcome of MoS_2_. By tuning the effective precursor dose during the ALD step, we drive the conversion outcome from isolated flakes to porous monolayers and ultimately to continuous monolayers with reduced sulfur vacancies. Comparative analysis of the oxide state, intermediate sulfurization state, and final electronic structure reveals that the optimized continuous monolayer exhibits improved stoichiometry, a lower Fermi‐level position, and reduced free‐charge screening. More importantly, these results show that the favorable low‐screening piezoelectric monolayer state is established during growth and sulfurization, rather than being achieved afterward through defect passivation or post‐growth healing. As a result, the resulting MoS_2_ monolayer delivers a competitive effective piezoelectric coefficient $d_{11}^{\mathrm{eff}}$ of 4.3 pm V^−1^, together with high device‐level sensitivity, and enables proof‐of‐usability demonstrations in wearable sensing as well as closed‐loop slippage feedback control.

## 2 Results and Discussion

### 2.1 Oxide‐Film Governs the Sulfurization Pathway and Final Monolayer Regime

A typical ALD process for ultrathin films usually involves adsorption, nucleation, and subsequent film growth. Although ALD is often considered a self‐limiting layer‐by‐layer growth process within a suitable temperature window, achieving continuous sub‐nanometer films remains challenging because nucleation delay and limited surface diffusion can still promote island formation and morphological nonuniformity in the ultrathin regime [9]. Such behavior makes precise control over continuity and thickness especially difficult for monolayer‐scale growth.

**FIGURE 1:**
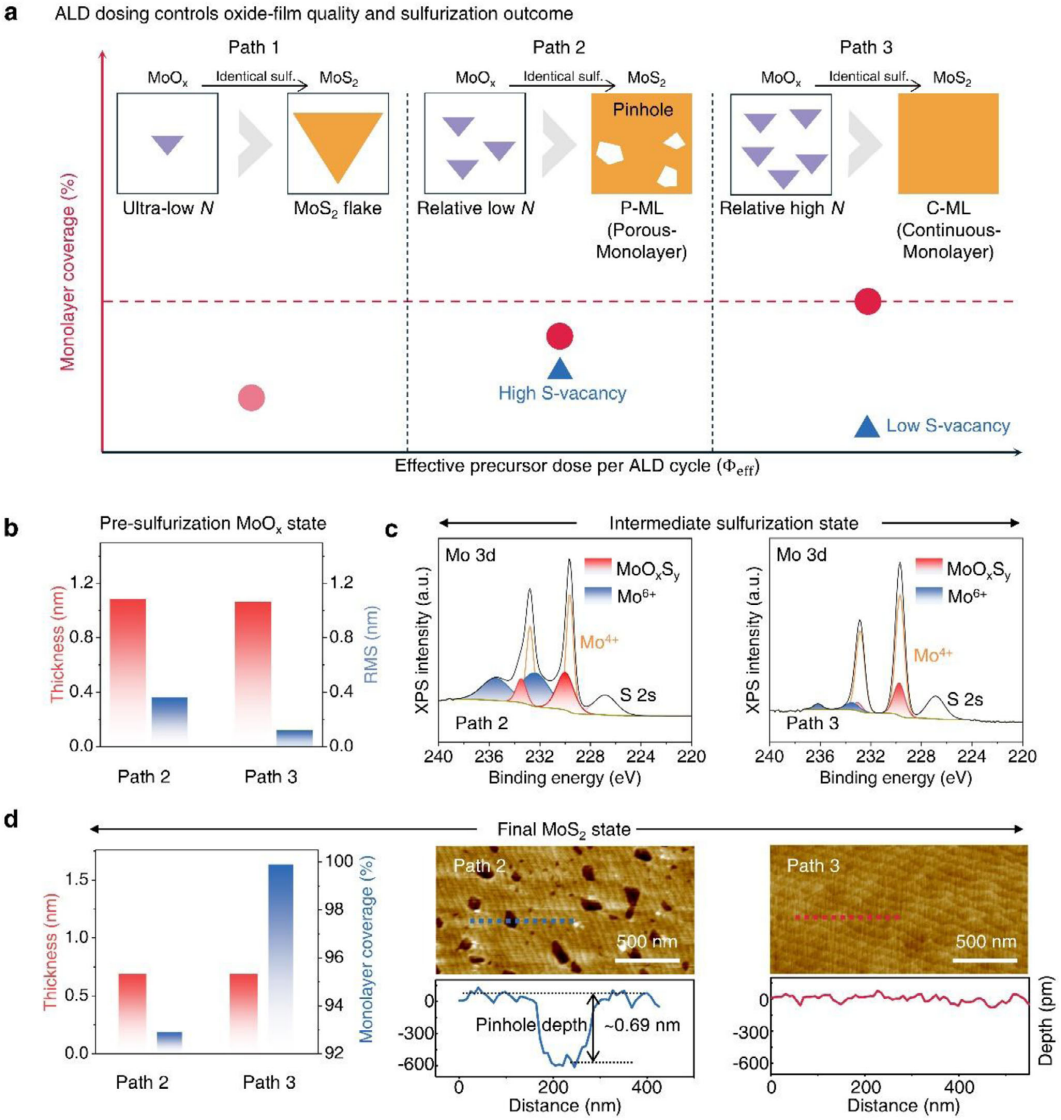
ALD dosing controls the evolution from the as‐deposited MoO_x_ film state to the final sulfurized MoS_2_ film. (a) Schematic illustration of the proposed framework: increasing the effective precursor dose per ALD cycle (Φ_eff_) modulates the quality of the as‐deposited MoO_x_ film, which subsequently governs the sulfurization pathway and determines the final MoS_2_ morphology and sulfur vacancy. Path 1 yields isolated MoS_2_ flakes at ultra‐low *N*, whereas Path 2 and Path 3 produce porous monolayer (P‐ML) and continuous monolayer (C‐ML) films, respectively, under identical sulfurization conditions. (b) Comparison of the pre‐sulfurization MoO_x_ film state for Path 2 and Path 3, showing similar film thickness but markedly different RMS roughness, with Path 3 exhibiting a smoother as‐deposited oxide film. (c) High‐resolution Mo 3d XPS spectra of the stepwise sulfurization intermediates collected after sulfurization at 600°C for 10 min, comparing the sulfurization states of the P‐ML and C‐ML samples under identical conditions. (d) Final MoS_2_ film comparison, including thickness/monolayer coverage statistics and representative AFM images with corresponding line profiles. Path 2 exhibits a pinhole‐rich porous monolayer with a local pinhole depth of about 0.69 nm, whereas Path 3 forms a nearly continuous monolayer with substantially higher coverage.

To address this challenge, we evaluate a two‐step route consisting of ALD deposition of MoO_x_ followed by sulfurization, in which the ALD step establishes the oxide‐film state and the subsequent sulfurization converts it into monolayer MoS_2_ (Figure S1). As illustrated in Figure 1a, increasing the effective precursor dose per ALD cycle modulates the quality of the as‐deposited MoO_x_ film, which subsequently governs the sulfurization pathway and determines the final MoS_2_ morphology under otherwise identical sulfurization conditions (Figure S2 and Table S1 describe how the ALD dose was adjusted). Under this fixed sulfurization condition, distinct growth outcomes are obtained by tuning the ALD dosing scheme. At low effective dose, the isolated MoS_2_ flakes are formed after sulfurization (Path 1), whereas intermediate and higher doses lead to a porous monolayer (P‐ML, Path 2) and a continuous monolayer (C‐ML, Path 3), respectively, with Path 2 exhibiting a relatively higher sulfur‐vacancy level than Path 3, as outlined in Figure 1a. Tables S1 and S2 shows the different parameters to deposit P‐ML and C‐ML (see experimental details in Methods).

To identify the origin of these distinct outcomes, we first compare the pre‐sulfurize MoO_x_ films in Figure 1b (see Note S1 for the experimental rationale and Note S2 for the detailed oxide‐film characterization in the Supporting Information). Although Path 2 and Path 3 show comparable oxide thickness before sulfurization (1.082 ± 0.016 nm vs 1.064 ± 0.028 nm), their as‐deposited oxide‐films differ in both surface morphology and chemical state, with Path 3 exhibiting a smoother oxide surface (RMS 0.12 nm vs 0.36 nm) and a more stoichiometric oxide composition. This comparison indicates that the divergence between P‐ML and C‐ML does not arise simply from oxide‐film thickness, but from differences in the oxide‐film state established during the ALD step.

We then tracked the conversion pathway through the intermediate sulfurization state (defined at 10 min). As shown in Figure 1c, the stepwise Mo 3d XPS spectra collected from the films after sulfurization at 600°C for 10 min under identical conditions reveal distinct sulfurization states for the Path 2 and Path 3 samples. Peak fitting of the Mo 3d and S 2p spectra sulfurized at 600°C for 10 min further supports distinct sulfurization states for the two samples under identical treatment conditions (the full peak‐fitted spectra at 5 and 10 min are provided in Figure S3). Specifically, the quantitative Mo 3d analysis (sulfurized at 600°C for 10 min) in Table S3 shows that the Mo^4+^ fraction increases from 0.37 in P‐ML to 0.75 in C‐ML, while the residual Mo^6+^ fraction decreases from 0.40 to 0.05, indicating a higher degree of sulfurization in Path 3. These observations indicate that subtle differences in the as‐deposited oxide film persist into the intermediate stage of sulfurization and propagate into distinct sulfurization conversion behaviors.

These differences ultimately manifest into the final MoS_2_ films (after sulfurization at 600°C for 60 min) as shown in Figure 1d. Despite their similar final thickness, Path 2 yields a pinhole‐rich porous monolayer with a local pinhole depth of about 0.69 nm, whereas Path 3 forms a nearly continuous monolayer with substantially higher monolayer coverage.

This oxide‐mediated MoS_2_ growth mechanism is further supported by the temperature‐ and dose‐dependent experiments in Figures S4 and S5, where monolayer coverage increases with both growth temperature and effective ALD dose. These results reinforce that precursor adsorption and dosage during ALD are critical for defining the intermediate oxide‐film state that governs the eventual sulfurization outcome. Detailed ALD dosing control methods are shown and discussed in Methods and Note S1 (See Figures S6–S8). Comparison between oxide films from Path 2 and Path 3 are shown in Note S2. More importantly, they show that the final monolayer regime is not dictated solely by sulfurization conditions, but is preconditioned by a growth‐history‐dependent oxide‐film state established upstream during ALD. Hence, the role of ALD dosing in the present system is not limited to routine process optimization; rather, it acts as a means of encoding the oxide‐to‐monolayer conversion pathway and, consequently, the piezoelectricity‐relevant final state.

### 2.2 Comparative Defect Analysis of Porous and Continuous Monolayer MoS_2_

**FIGURE 2:**
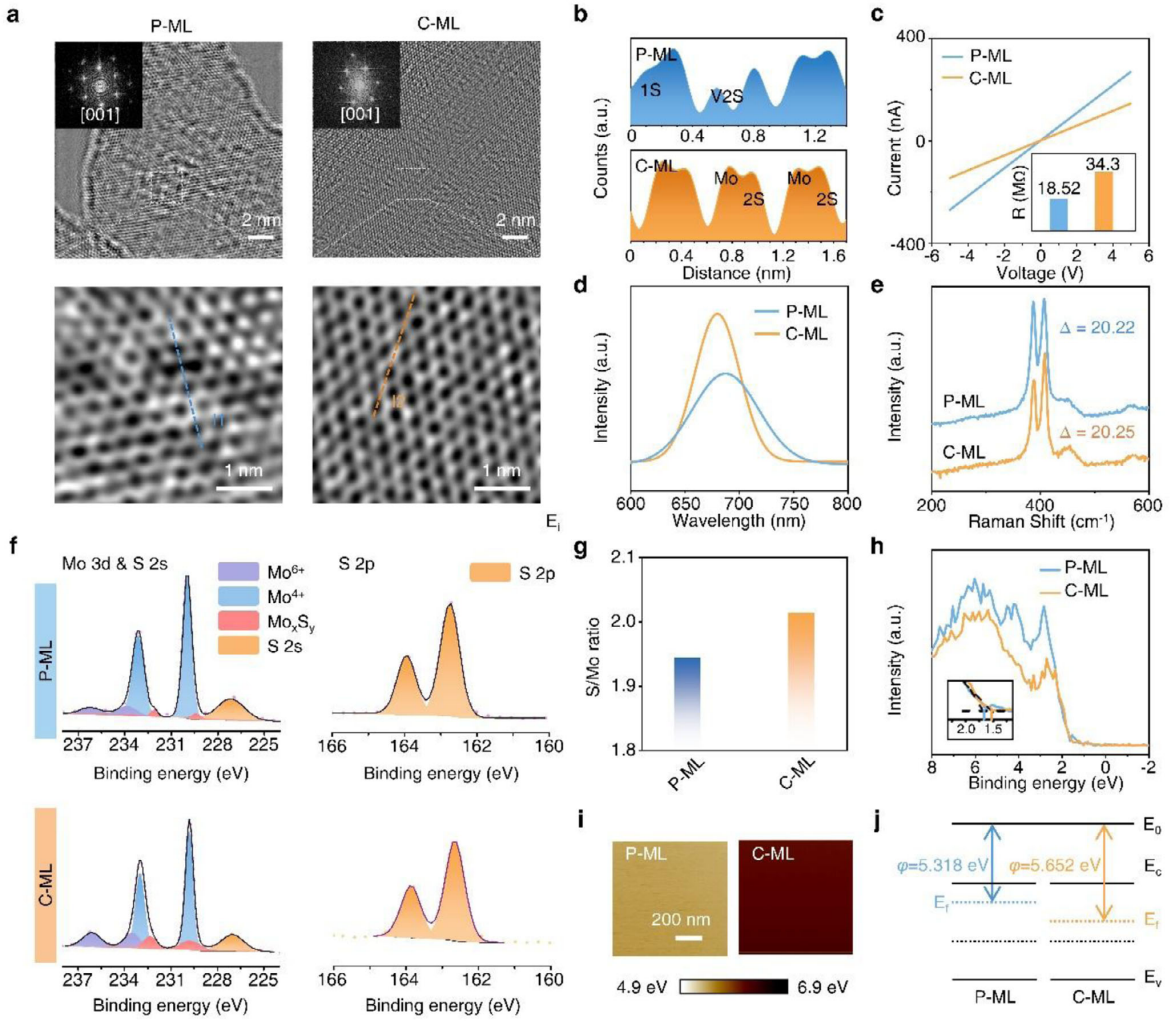
Structural, chemical, and electronic characterization of vacancy‐rich (P‐ML) and vacancy deficient (C‐ML) monolayer MoS_2_. (a) High‐resolution STEM images and corresponding fast Fourier transform (FFT) patterns (insets) of P‐ML and C‐ML MoS_2_, highlighting differences in defect density. The lower panels show atomic‐resolution images illustrating local lattice structures. (b) Line‐scan profiles in (a), revealing differences in defect density between P‐ML and C‐ML. (c) I‐V curves of P‐ML and C‐ML, with an inset quantifying the resistance, demonstrating enhanced lower carrier density in C‐ML. (d) Photoluminescence spectra of P‐ML and C‐ML, indicating differences in optical quality and defect‐related nonradiative recombination. (e) Raman spectra of P‐ML and C‐ML, showing characteristic E_2g_ and A_1g_ modes and their respective peak positions, reflecting variations in strain and defect content. (f) XPS spectra of Mo 3d, S 2s, and S 2p regions for P‐ML and C‐ML, illustrating differences in chemical composition, oxidation states, and stoichiometry. (g) S/Mo atomic ratios for P‐ML and C‐ML, confirming improved stoichiometric control in C‐ML. (h) Valence band XPS spectra, highlighting the shift in valence band maximum (VBM) between P‐ML and C‐ML (inset quantifies the energy difference). (i) KPFM work function mapping of P‐ML, C‐ML films, demonstrating a difference in work function. (j) Schematic energy band diagrams for P‐ML and C‐ML.

To elucidate the structure‐defect‐property relationship in monolayer MoS_2_, we systematically compare porous monolayers (P‐ML, from path 2) and continuous monolayers (C‐ML, from path 3) using advanced structural and spectroscopic characterization (Figure 2). High‐resolution transmission electron microscopy (HRTEM) images (Figure 2a) reveal the hexagonal lattice structures of both P‐ML and C‐ML, with corresponding FFT patterns shown in the insets. Notably, C‐ML exhibits superior film continuity (coverage ∼99.97%) compared to P‐ML (∼92.9%). Both P‐ML and C‐ML demonstrate polycrystalline features, as evidenced by the corresponding FFT patterns in Figure S9. The High‐angle annular dark‐field scanning TEM (HAADF‐STEM) images further indicate that C‐ML possesses a significantly lower defect density than P‐ML (Bottom in Figure 2a,b). In P‐ML, the observed atomic features are consistent with sulfur‐vacancy‐related defects, such as V_S_ or V_2S_, indicating a higher defect density than in C‐ML [11, 27, 28]. Electrical measurements (Figure 2c) show that P‐ML and C‐ML have resistances of 18.52 MΩ and 34.3 MΩ, respectively, under ohmic contact, consistent with the lower defect density and higher quality of C‐ML deposited by Path 3 (Figure 1a). Photoluminescence (PL) spectra (Figure 2d) demonstrate that C‐ML exhibits a much sharper emission peak (FWHM = 85.87 nm) compared to P‐ML (FWHM = 281.94 nm), further evidencing the higher crystalline quality of C‐ML, which is critical for piezoelectric applications (see Section 2.3). Raman spectroscopy (Figure 2e) confirms the monolayer nature of both films, with characteristic peak separations of 20.22 cm^−1^ for P‐ML and 20.25 cm^−1^ for C‐ML. Additionally, C‐ML displays a blueshifted Raman peak relative to P‐ML, likely due to compressive strain retained in the continuous film, whereas the abundant pinholes in P‐ML facilitate strain relaxation. Raman mapping (Figure S10) further reveals poorer uniformity in P‐ML.

X‐ray photoelectron spectroscopy (XPS, Figure 2f and Figure S11) reveals distinct chemical states for the two films. In the Mo 3d region, C‐ML shows Mo^4+^ 3d_3/2_ and 3d_5/2_ peaks at 232.97 and 229.82 eV, alongside S 2p_1/2_ and S 2p_3/2_ peaks at 163.85 and 162.6 eV, all slightly lower than those of P‐ML. This binding energy shift is consistent with a downward shift of the Fermi level toward the valence band in C‐ML [10]. Stoichiometric analysis (Figure 2g) shows S/Mo ratios of 1.945 for P‐ML, lower than those of C‐ML (2.014), consistent with a higher sulfur‐vacancy concentration in the porous samples. In addition, the valence band (VB) XPS (Figure 2h) further supports a lower defect density and carrier concentration in C‐ML, consistent with its intrinsic n‐type character. Kelvin probe force microscopy (KPFM, Figure 2i) reveals a higher work function for C‐ML (5.652 eV) than for P‐ML (5.318 eV), indicating a lower Fermi‐level position in C‐ML. Considering the intrinsic n‐type character of monolayer MoS_2_, this work‐function increase is consistent with reduced electron accumulation and weaker free‐charge screening in C‐ML. In combination with the improved S/Mo stoichiometry and the valence‐band XPS results, these data support that the continuous monolayer contains fewer vacancy‐related donor states and therefore preserves the piezoelectric polarization more effectively. The schematic band diagram in Figure 2j summarizes the corresponding electronic‐structure difference between P‐ML and C‐ML. To verify that the KPFM‐observed surface‐potential difference is representative rather than a local effect, large‐area Kelvin probe measurements were further carried out on both films (See Figure S27). The Kelvin probe results reproduce the same trend as the KPFM data, consistent with a lower Fermi‐level position and minimized screening of piezoelectric charges in C‐ML [10, 25, 29]. Overall, these results demonstrate clear differences in defect density, chemical composition, and electronic structure between P‐ML and C‐ML, which provide the structural and electronic basis for their distinct piezoelectric responses discussed in Section 2.3.

### 2.3 Piezoelectric Performance

**FIGURE 3:**
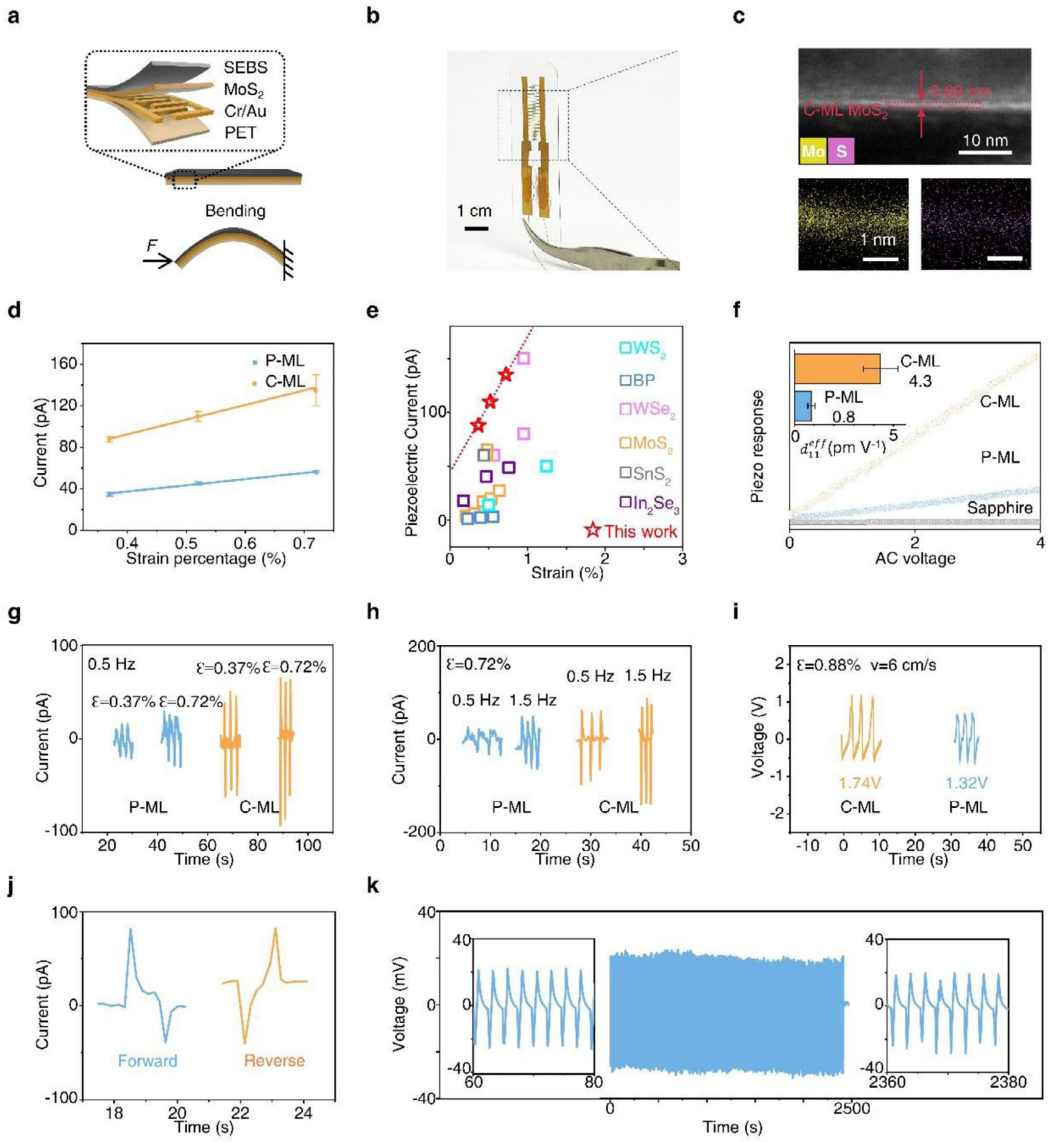
Piezoelectric performance of P‐ML and C‐ML MoS_2_ films. (a) Illustration of the flexible piezoelectric device architecture, featuring MoS_2_ encapsulated with SEBS on a PET substrate. (b) Photograph of the flexible piezoelectric device based on the engineered MoS_2_ monolayer. (c) High‐resolution STEM image and elemental mapping of the C‐ML MoS_2_. (d) Piezoelectric current as a function of applied strain percentage for P‐ML and C‐ML. (e) Benchmark plot of piezoelectric current versus strain for various 2D materials, highlighting the superior performance of the C‐ML MoS_2_ achieved in this work compared to previously reported binary TMDs, details in Table S4. (f) Piezoresponse force microscopy (PFM) measurements of P‐ML and C‐ML. (g) Strain‐dependent piezoelectric response. (h) Frequency‐dependent piezoelectric response. (i) Output voltage signals from C‐ML and P‐ML with a bending strain of 0.88% and a bending velocity of 6 cm s^−1^. (j) Comparison of forward and reverse piezoelectric current response for a C‐ML device, demonstrating the polarity of the generated current. (k) Long‐term cycling stability of the piezoelectric voltage output from a C‐ML device under continuous cyclic bending over an extended period (2500 s, 1000 cycles), with insets showing magnified views of initial and final cycles, confirming excellent durability (strain: 0.37%, frequency: 0.5 Hz).

To compare the piezoelectric performance of P‐ML and C‐ML, we integrated the films into flexible devices and evaluated their electromechanical responses under in‐plane 1–1 mode strain [6, 30]. As illustrated in Figure 3a, the device consists of a monolayer MoS_2_ film transferred onto a polyethylene terephthalate (PET) substrate, contacted by Cr/Au electrodes, and encapsulated with styrene‐ethylene‐butylene‐styrene (SEBS) for mechanical robustness. The transferred film retains good integrity after the wet‐transfer process, as shown in Figure S12. Figure 3b shows the integrated piezoelectric sensor fabricated using C‐ML, whose atomic‐scale thickness of approximately 0.69 nm is confirmed by cross‐sectional STEM imaging (Figure 3c). Controlled bending was applied to induce well‐defined lateral strain in the devices. Both P‐ML‐ and C‐ML‐based devices exhibit an approximately linear piezoelectric response over the tested strain range up to 0.72% (Figure 3d). The strain calculation used in the device measurements is provided in Figure S13. Notably, the C‐ML device demonstrates a sensitivity of 141.38 pA per 1% lateral strain at 0.5 Hz, which is 2.36 times higher than the 59.69 pA per 1% lateral strain observed for P‐ML (Figure 3d). Benchmarking against previously reported binary TMD piezoelectric sensors shows that the C‐ML device delivers the highest current and voltage sensitivity among the compared binary TMD systems, as summarized in Figure 3e and Table S4 [6, 10, 26, 30, 31, 32, 33, 34, 35, 36]. Additional voltage output and sensitivity data of C‐ML at different bending strains are provided in Figure S14. To probe the effective piezoelectric properties, Piezoresponse Force Microscopy (PFM) was performed, revealing $d_{11}^{\mathrm{eff}}$ coefficients of 4.3 pm V^−1^ for C‐ML and 0.8 pm V^−1^ for P‐ML (Figure 3f). Notably, the $d_{11}^{\mathrm{eff}}$ obtained for C‐ML is competitive within the reported monolayer MoS_2_ piezoelectric literature [25, 37]. This substantial enhancement in $d_{11}^{\mathrm{eff}}$ for C‐ML is consistent with its higher crystalline quality, lower vacancy density, and reduced charge screening discussed in Section 2.2, supporting that the favorable low‐screening monolayer state established through growth and conversion can translate into enhanced electromechanical functionality. The integrated devices also exhibit pronounced strain‐ and frequency‐dependent current outputs, with clear signal generation over a strain range of 0.20% to 0.72% (corresponding to bending radii of ∼24.25 and ∼6.7 mm, respectively, the calculation details as shown in Figure S13a with PET thickness of ∼97 µm) and frequencies from 0.5 to 1.5 Hz (Figure 3g,h). Such robust electromechanical coupling highlights the potential of these monolayer MoS_2_ films for high‐performance piezoelectric sensing applications. Furthermore, under dynamic bending at a velocity of 6 cm s^−1^ and a strain of 0.88%, the open‐circuit voltage output reaches 1.74 V for C‐ML and 1.32 V for P‐ML (Figure 3i), indicating their potential in piezoelectric energy nanogenerators (PENGs). To confirm the piezoelectric origin of the output, polarity‐reversal tests were conducted, showing that the current signals invert upon reversing the wiring direction while maintaining a comparable amplitude (Figure 3j). The presence of strain in MoS_2_ films during substrate bending or stretching was further supported by Raman and PL measurements, which revealed characteristic spectral shifts corresponding to the applied mechanical deformation (Figure S15). Long‐term durability testing further reveals that the C‐ML device maintains stable signal output after 2500 s of cyclic bending at 0.37% strain and 0.5 Hz (Figure 3k), demonstrating excellent mechanical and electrical reliability.

The C‐ML/P‐ML piezoelectric ratio drops from ∼5 at the microscale to ∼2.3 at the macroscale (Figure S16). Figure S16a illustrates that defects can exert competing effects on the electromechanical response: local strain asymmetry may enhance the response, whereas charge screening can suppress it. Consistent with the finite‐element analysis in Figure S16b, the abundant pinholes in P‐ML can generate localized strain gradients, which may partially compensate for its lower intrinsic piezoelectricity at the device scale [38, 39]. Thus, even though C‐ML shows lower screening and a higher $d_{11}^{\mathrm{eff}}$, the unique strain landscape in P‐ML partially compensates at the macroscale. These results show that the superior effective piezoelectricity and stable device output of C‐ML make it suitable for the practical demonstrations discussed in the following section.

### 2.4 Practical Applications of C‐ML MoS_2_: Biocompatibility, Human‐Motion Sensing, and Intelligent Feedback Control

**FIGURE 4:**
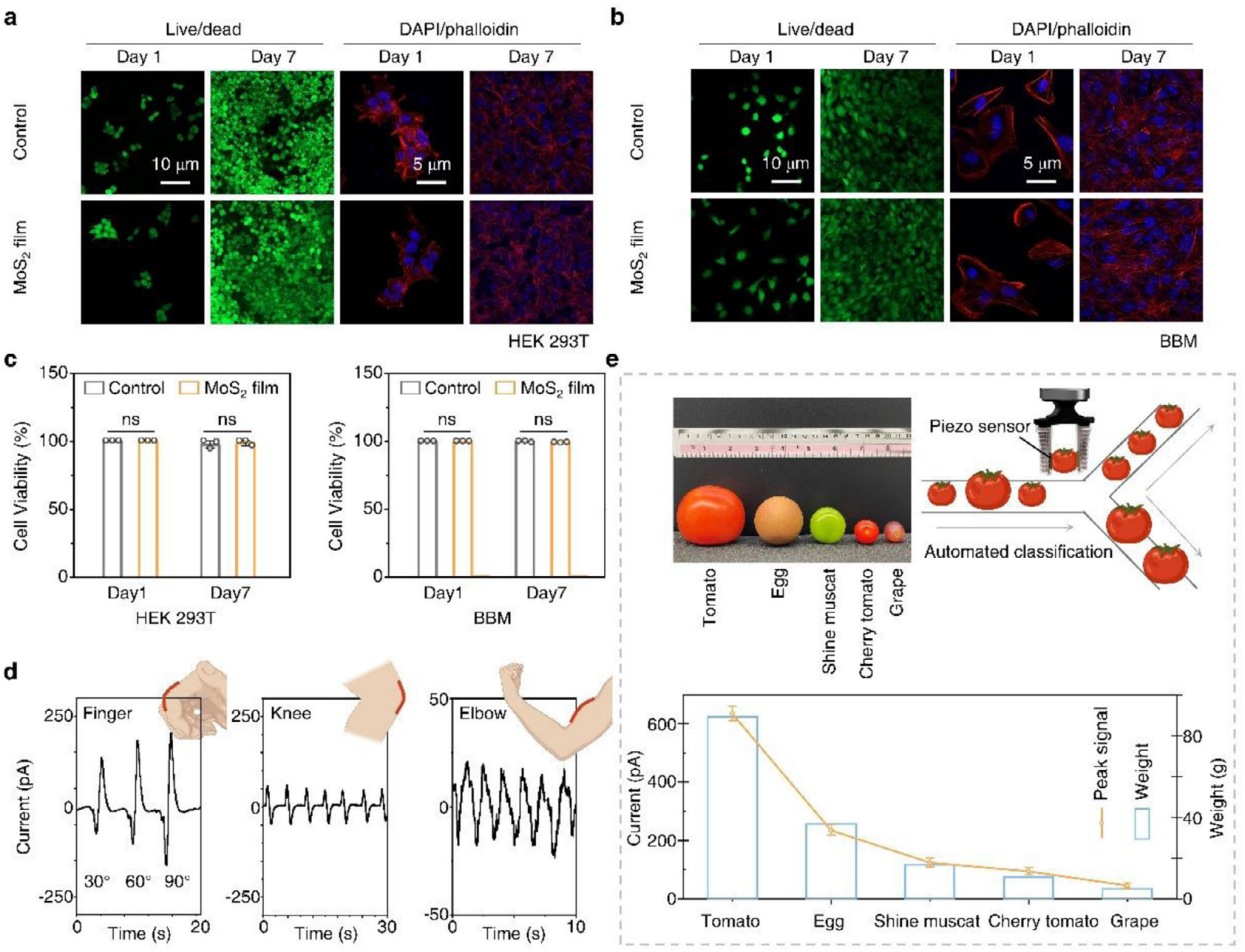
Biocompatibility assessment and demonstration of practical applications of engineered MoS_2_‐based piezoelectric devices. (a,b) Representative images of live/dead staining (Green and red labels indicate living and dead cells, respectively) and DAPI/ phalloidine staining (red and blue labeled cells represent the cytoskeleton and nucleus of cells, respectively) of HEK 293T and BBM cells following incubation with MoS_2_ film. (c) Viability of HEK 293T and BBM cells after incubation with MoS_2_ film. (d) Demonstration of real‐time monitoring of physiological motions including finger bending angle sensing and body motion sensing at finger, elbow, and knee (IRB‐2024‐015). (e) Demonstration of fruit automated classification by sensing the weight of fruits.

Given its superior piezoelectric performance, mechanical stability, and film continuity, C‐ML was further explored for biocompatibility and practical sensing applications. First, we conducted a comprehensive in vitro biocompatibility assessment of C‐ML by culturing it with two cell lines, including human embryonic kidney (HEK 293T) cells and human bronchial epithelium (BBM) cells. Live/dead assays, along with 4′,6‐diamidino‐2‐phenylindole (DAPI) and phalloidin staining, were performed at 1, 3, and 7 days post‐culture to evaluate cell viability and morphology. Nearly all cells cocultured with C‐ML exhibited strong green fluorescence comparable to the media‐only control, indicating high cell viability (Figure 4a,b and Figure S17). In addition, cells cultured on C‐ML showed spreading behavior and filopodia extension similar to those of the control group. Therefore, these results undoubtedly confirmed that these MoS_2_ films were of great biocompatibility. Quantitative analysis further confirmed that cell viability remained above 98.8% for both cell types throughout the culture period, with no significant differences between the C‐ML and control groups (Figure 4c). C‐ML‐based piezoelectric sensors were then applied to real‐time human motion monitoring. The devices reliably detected bending motions at the finger, knee, and elbow joints, generating stable and reproducible electrical signals (Figure 4d). This demonstrates the potential of C‐ML‐based devices for wearable health monitoring and human–machine interface applications. Furthermore, C‐ML piezoelectric sensors were integrated into a soft robotic system for automated object classification based on weight. As illustrated in Figure 4e, the sensor output varied systematically with the weights of different fruits, enabling automated fruit classification. This highlights This result highlights the potential of C‐ML sensors for intelligent soft‐robotic and Internet of Things (IoT) applications.

**FIGURE 5:**
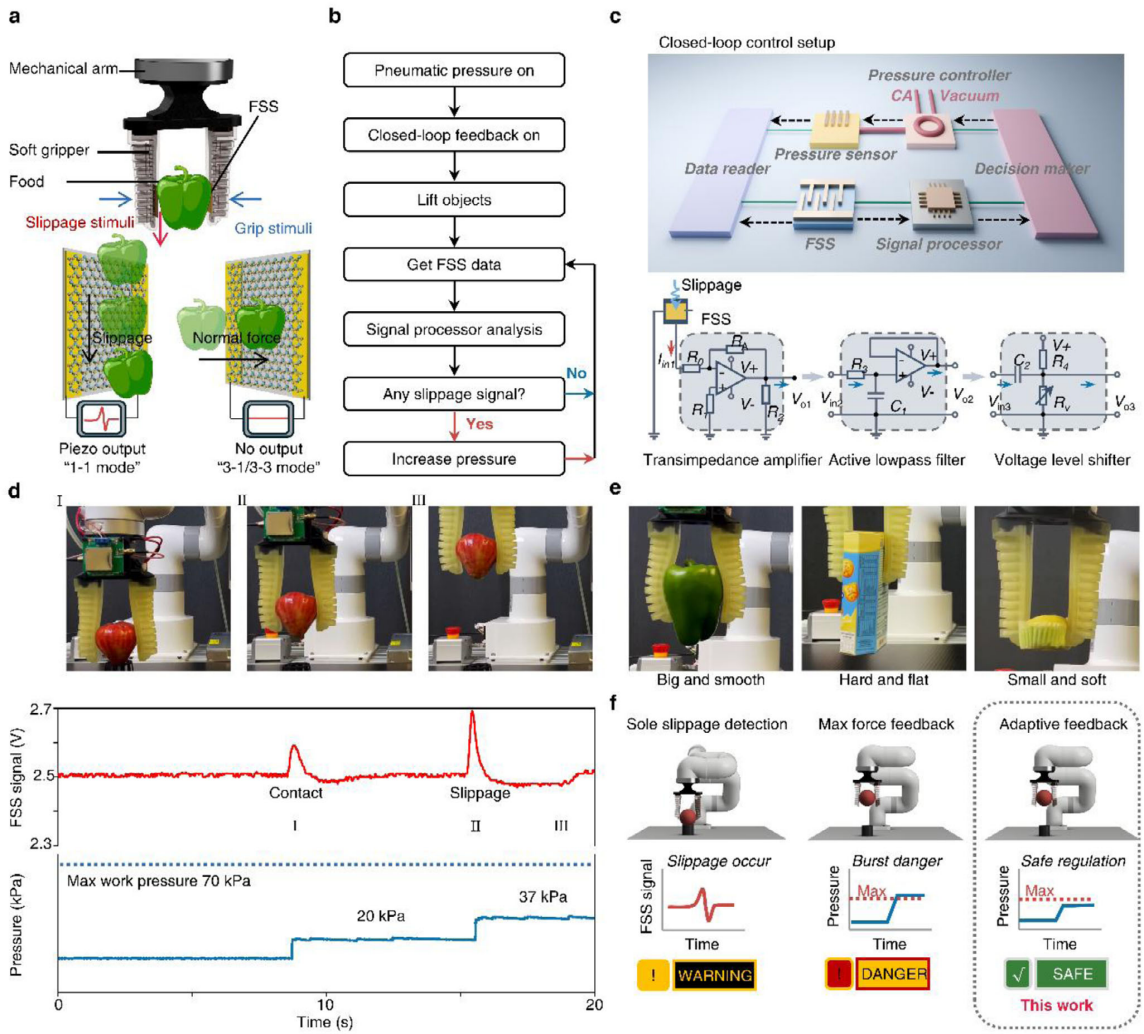
Demonstration of closed‐loop slippage feedback control for soft robotics. (a) Illustration of a pneumatic gripper equipped with a flexible slippage sensor (FSS) based on an engineered MoS_2_ piezoelectric device, which is sensitive to slippage instead of normal force. (b) Algorithm diagrams of the slippage feedback closed‐loop control system. (c) Illustration of closed‐loop controller, including pressure sensor, pressure controller, FSS, signal processor, data reader, and decision maker. Inset represents the simplified equivalent circuits for signal processors, consisting of a transimpedance amplifier, active lowpass filter, and voltage level shifter. (d) Photos and signals from sensors during a slippage feedback closed‐loop control event. (e) Versatility of the closed‐loop feedback system for interaction between diverse objects. (f) Comparison between sole slippage detection, maximum force feedback, and adaptive pressure feedback.

Piezoelectric sensors based on MoS_2_ exhibit greater sensitivity to tangential (shear) forces compared to normal (tapping) forces, enabling precise detection of slippage events during object manipulation (Figure 5a). Compared with the preceding wearable‐sensing demonstrations, this slippage feedback control experiment represents the more functionally significant application in the present work because it requires rapid detection of subtle interfacial disturbances together with timely grip adjustment. Inspired by the human tactile feedback system, we developed an on‐demand closed‐loop slippage feedback control system integrated with a soft robotic gripper. This system delivers real‐time slippage signals to the gripper controller, allowing for adaptive adjustment of the gripping force to prevent object loss (Figure S18), governed by a built‐in algorithm (Figure 5b). In the present system, this capability is achieved mainly through the intrinsic electromechanical response of the optimized monolayer MoS_2_ platform, rather than through deliberately engineered mechanical amplification structures or complex tactile array architectures. The system architecture comprises a robotic arm equipped with a double‐finger soft gripper (fabricated by 3D‐printing), a flexible slippage sensor (FSS), and a closed‐loop controller (Figure 5c). To ensure robust and accurate signal transmission, the FSS output is processed via a custom signal processor that amplifies the sensor signal, filters background noise, and adjusts the baseline offset (Figure 5c). Figure 5d demonstrates the real‐time adaptive regulation by this system: upon slippage detection after initial contact, the gripping pressure is rapidly increased from 20 to 37 kPa within approximately 250 ms, successfully preventing object drop (Video S1). In contrast, lifting attempts without feedback control result in failed grasping, as shown in Figure S19. The adaptive pressure regulation was further validated across a range of objects with varying shapes and mechanical properties, confirming broad compatibility and operational robustness (Figure 5e and Figure S20 and Videos S2 and S3). It is noteworthy that the maximum design pressure for the soft gripper is 70 kPa. Beyond simple slippage detection, this system enables adaptive feedback control, dynamically modulating the gripping force rather than applying maximum pressure immediately upon slippage (Figure 5f and Figure S21 and Video S4). This strategy provides safer and more intelligent gripping by reducing the risk of damage to either the gripper or the object while maintaining secure handling. The adaptive control performance is consistent with the stable piezoelectric output of the C‐ML device together with the rapid response of the integrated control circuit.

## 3 Conclusion

In conclusion, we have demonstrated a controllable strategy to tailor the morphology, defect density, and piezoelectric performance of monolayer MoS_2_ by regulating the effective ALD dose during oxide‐film deposition. By tuning the ALD dose, the quality of the as‐deposited oxide film is modulated, which in turn affects the subsequent sulfurization pathway and ultimately determines the film continuity and defects of the final MoS_2_ film. This process enables the evolution from isolated flakes to porous monolayers and finally to continuous MoS_2_ monolayers. The optimized continuous monolayer exhibits lower sulfur vacancy density and enhanced piezoelectric performance, delivering a competitive $d_{11}^{\mathrm{eff}}$ of 4.3 pm V^−1^ together with high voltage and current sensitivities under mechanical deformation. More importantly, this favorable piezoelectricity‐relevant monolayer state is established preventively during growth and oxide‐to‐monolayer conversion, rather than recovered afterward through defect passivation or post‐treatment after defect emergence. In addition, the film shows good biocompatibility, mechanical reliability, and stable device output, enabling applications in human‐motion monitoring, fruit classification, and closed‐loop slippage feedback control. These findings provide a clear correlation between ALD dose, oxide‐film quality, sulfurization behavior, and final device performance, offering useful guidance for the design of high‐performance flexible piezoelectric devices based on two‐dimensional materials.

## 4 Methods

### 4.1 Atomic Layer Deposition

Single‐side polished *c*‐plane sapphire substrates with a size of 1 cm by 1 cm were purchased from Suzhou Crystal Silicon Electronic & Technology. The substrates were sequentially ultrasonic cleaned in acetone, ethanol, and deionized water for 10 mins each, followed by drying using compressed air. Oxygen plasma treatment (IoN 40 plasma system, PVA Tepla) was applied to substrates to promote the deposition, with the power of 300 W, O_2_ flow rate of 300 sccm, and duration of 10 mins, before ALD process. Mo(CO)_6_ (99.99%, Sigma–Aldrich) was used as the precursor for the deposition of MoO_x_ during the ALD process. Ozone generated at 1.0 mbar partial pressure using an ozone RF generator (Kaimei lab‐II) was applied as the co‐reactant with a flow rate of 200 sccm for all the ALD processes. High‐purity nitrogen was acting as carrier gas and purging gas with a continuous flow rate of 50 sccm to maintain a base pressure of around 50 Pa. To prevent precursor condensation, all the tube lines were heated to 120°C. The chamber temperature was set as 150°C–180°C. Prior to deposition, chamber temperature and tube lines temperature were held for 2 hrs. During the ALD process, vaporized precursor (held in a bubbler at room temperature) and ozone were alternatively and repeatedly pulsed until the assigned cycle number was reached. Table S1 lists the recipes for Path 1 to Path 3, including the dose, holding, and purge times. The parameters to deposit P‐ML and C‐ML are shown in Tables S1 and S2.

The sulfurization process is fixed to all samples in this work to study the effect of ALD kinetic flux on the produced MoS_2_. Sulfur powder was used as the sulfur source to convert the deposited MoO_x_ film to a MoS_2_ film. The MoO_x_/sapphire samples were placed in the middle of a tube furnace with the support of a crucible, while 500 mg of sulfur powder (99.99%, Sigma–Aldrich) was placed upstream in the tube. Before sulfurization, the tube was maintained under a vacuum of around 1 mbar under a temperature of 120°C for 1 h, followed by purging using high‐purity argon for 5 times, to remove moisture and organic contamination. The temperatures of the sample and sulfur were held at 600°C and 150°C for 1 h, followed by 900°C and 200°C for 30 min to further enhance the crystallinity of MoS_2_. A high‐purity argon gas flowed from upstream to carry the sulfur vapor and prevent unwanted oxidation, with a flow rate of 80 sccm. After the sulfurization, the furnace was cooled to room temperature for over 3 h.

### 4.2 Device Fabrication

The MoS_2_ films were transferred from the as‐grown substrates to target substrates (rigid or flexible) using a wet transfer process. Specifically, a Polymethyl metaxrylate (PMMA, Kayaku Advanced Materials) protective layer (4% in anisole, MW of PMMA: 495 000) with a thickness of ∼200 nm was spin‐coated onto the MoS_2_/sapphire sample, followed by heating at 150°C for 3 min to form a PMMA film. The samples were then floated on a KOH solution (2 m in DI‐water) for 6 h to etch away the sapphire layer beneath the MoS_2_ film, allowing separation between the MoS_2_ film and the substrate. Subsequently, the etched films were cleaned in DI water for 30 min. Cleaned new substrates, such as PET or Silicon wafer, were used to pick up the floating film, which was then dried at 70°C for 5 min. To remove the surface PMMA, the transferred samples were immersed in acetone for 10 min.

A commercial PET sheet (3 m) with a thickness of 94 µm was used as the bottom substrate for the bendable piezoelectric device. The PET sheets were ultrasonic cleaned in ethanol and DI‐water for 10 min each. Thermal evaporation (Kurt J. Lesker PVD75) was employed to pattern Cr/Au electrodes on the PET sheets, with electrode thicknesses of 5 nm for Cr and 50 nm for Au. The width and distance between adjacent finger electrodes were both 500 µm. Afterward, the single‐layer MoS_2_ films were transferred onto PET substrates with integrated electrodes. To prevent unintended damage and potential oxidation of the MoS_2_ active layer, a protective polymer, Styrene Ethylene Butylene Styrene copolymer (SEBS, Tuftec H1052) layer with a thickness of approximately 10 microns was spin‐coated to cover the MoS_2_.

### 4.3 Characterizations of Materials and Integrated Devices

A commercial AFM system (Dimension Icon, Bruker) was utilized to determine the surface morphologies of the deposited films, while Kelvin probe force microscopy (KPFM) modes were conducted to measure work function with the aid of a conductive SCM‐PIT tip that is coated by Pt/Ir. Large‐scale Kelvin Probe surface potential mapping are collected using a scanning Kelvin probe system (SKP5050, KP Technology). Lateral piezoresponse force microscopy (LPFM) measurements were performed using a Bruker Dimension Icon atomic force microscope with a Pt/Ir‐coated conductive SCM‐PIT probe (Bruker; nominal spring constant of 3 N m^−1^ and nominal resonance frequency of 75 kHz). To generate an in‐plane electric field across the sample, lateral Au electrode pairs with a gap of 10 µm were fabricated on the substrate, and the AC excitation voltage was applied across the electrodes. To reduce resonance‐related artifacts, the excitation frequency was fixed at 15 kHz, well below the tip‐sample contact resonance frequency. For quantitative analysis, the normal deflection sensitivity was first calibrated from the slope of the force‐distance curve acquired in contact mode on a hard and flat sapphire substrate. The in‐plane sensitivity (*S*_IP_) used for LPFM quantification was then derived from the calibrated out‐of‐plane sensitivity (*S*_OOP_) and the cantilever geometry following *S*_OOP_ = *S*_IP_  × 2*L*/3*h*, where *L* and *h* are the cantilever length and tip height, respectively [40]. The calibrated in‐plane sensitivity was used to convert the lock‐in output from voltage to displacement, and the effective in‐plane piezoelectric coefficient was extracted from the linear dependence of the background‐corrected LPFM displacement amplitude on the applied AC voltage (0–4 V). Because residual contributions from contact mechanics, electrostatic interactions, and geometry‐dependent transduction cannot be completely excluded, the extracted coefficient is conservatively reported as an effective in‐plane piezoelectric coefficient $d_{11}^{\mathrm{eff}}$.

Raman and photoluminescence spectra were employed on the WITec Alpha‐300 SR with a 488 nm laser with a spot size of 1 µm in air. A transmission electron microscope (TEM, JEM‐2100F) operating at an accelerating voltage of 200 kV was employed to obtain high‐resolution TEM images (HR‐TEM). The elemental distributions were studied using high‐angle annular dark‐field scanning TEM (HAADF‐STEM) and corresponding windowless energy dispersive X‐ray (EDX) spectroscopy (Oxford EDX detector) on the cross‐section of the C‐ML. A focused ion beam (FIB, Zeiss Crossbeam 540) was applied to prepare the cross‐sectional lamellae for TEM analysis. The top‐view TEM samples were prepared by wet transfer of MoS_2_ onto TEM grids. Film Thickness was measured on a Spectroscopic Ellipsometer (VASE VB‐400) at wavelengths of 300 to 1700 nm with an interval of 20 nm. An X‐ray photoelectron spectrometer (XPS, Shimadzu Kratos Axis Supra) was utilized to analyze the composition and chemical state with Al Kα radiation operating at 15 kV and 15 mA. The TGA measurement was performed on TA Instruments Q500. Approximately 16 mg of the precursor was loaded into an alumina crucible and heated at 20 K min^−1^ from 20 to 400 °C. Argon at a flow rate of 40 ml min^−1^ was used as the carrier gas. The *I*–*V* curves were measured by a Keithley 4200 Analyzer. The water contact angles on substrates with different treatments were measured using Dataphysics OCA 15EC. The bending and stretching tests were controlled by a linear actuator with a built‐in programmable controller and a 5 µm encoder (Elshin LDL25). The output voltage and current signals were measured by an electrometer (Keithley, 6514B) with 200 TΩ input impedance, and a picoammeter (Keithley, 6485) with an input impedance of 1 kΩ. This work was approved by the Institutional Review Board (IRB) at Nanyang Technological University (IRB‐2024‐015). All the experiments involving human motions were conducted with the agreement of volunteers.

### 4.4 Biocompatibility Assay

HEK 293T and BBM cells were purchased from the American Type Culture Collection (ATCC). Cells were cultured in DMEM medium with HEPES (Hyclone; Cat# SH30243.FS) supplemented with 10% fetal bovine serum (FBS; Thermo Fisher Scientific; Cat# 26140079), Penicillin‐Streptomycin (Thermo Fisher Scientific; Cat# 15140163. For both assays, 40,000 cells were plated in 12‐well plates and cocultured without or with MoS_2_ films for 1, 3, and 7 days. Live/dead assay at specified time points was assessed using the LIVE/DEAD Viability/Cytotoxicity Kit (Invitrogen; Cat# L3224). After washing with PBS 3 times, the cells were stained with calcein‐AM/EthD‐1 according to the manufacturer's instruction. Then the cells were imaged using a laser confocal microscope (Zeiss LSM 880). For DAPI/phalloidin assay, after washing with PBS 3 times, the cells underwent fixation using a solution of 4% PFA and 2% sucrose, followed by six washes with PBS‐CM (1X PBS containing 1 mM Magnesium Chloride (MgCl_2_) and 1 mm Calcium chloride (CaCl_2_)). Subsequently, permeabilization was carried out in PBSCM‐T (PBSCM with 0.1% Triton X‐100). For blocking, cells were treated with FDB buffer (5% FBS, 5% goat serum, 2% BSA in PBS, 1 mm MgCl_2_, and 1 mm CaCl_2_) and then incubated with Alexa Fluor 546 Phalloidin (Invitrogen; Cat# A22283), both prepared in FDB buffer, for 1 h at room temperature. Extensive washing followed each antibody incubation. Coverslips were mounted using vectashield containing DAPI for nuclear staining. Observation and capture of images were performed using a Zeiss LSM 880 confocal laser scanning microscope.

### 4.5 Fabrication of Soft Gripper

The 3D printing of the pneumatic finger was performed using an Anycubic Photon DLP printer. The ink formulation for the printing process consisted of a mixture of Ebercyl 8413 and Ebercyl 113 in a 1:1 ratio [41]. To enable the curing process, photoinitiators were added: 1 wt.% TPO (Diphenyl(2,4,6‐trimethylbenzoyl) phosphine oxide), 1 wt.% BAPO (Bis(2,4,6‐trimethylbenzoyl)‐phenylphosphine oxide), and 0.2 wt.% MEHQ (4‐Methoxyphenol) as an inhibitor to stabilize the formulation. The printing process parameters were as follows: the first layer was exposed for 25 s to ensure proper adhesion to the build plate, while the subsequent layers were exposed for 13 s each. The Z layer height was set to 0.1 mm to achieve the desired resolution and print quality. The pneumatic finger design measured 72 mm in length and had a tapered height, decreasing from 20 mm at the base to 15.5 mm at the tip. The structure consisted of chambers, each 5 mm in width with a 1 mm gap between them, allowing for flexible movement and pneumatic actuation. The design of the soft finger is shown in Figure S22.

### 4.6 Slippage Feedback Closed‐Loop Control System

Two sets of solenoid valves were employed to regulate the flow of compressed air (CA) and vacuum within the distribution line. A differential pressure sensor (MPX5100DP) was utilized to monitor the pressure within the distribution line through Arduino, controlling the opening and closing of the solenoid valve. The signal of the flexible slippage sensor (FSS) was processed by a transimpedance amplifier, an active lowpass filter, and a voltage level shifter in sequence. The signals from FSS (embedded onto the soft gripper) and the pressure sensor were transmitted to two Arduino units: one for serial recording and plotting, and the other for making decisions and autonomous feedback control. When the FSS signal exceeds the normal noise, ranging from 2.48 to 2.52 V, the controller applies a larger grip force by increasing the pressure with a step of 1 kPa until the FSS signal drops within the noise range.

## Author Contributions

Y.L., M.R.J.T., and P.S.L. conceived the idea and designed the experiments. Y.L. deposited the MoS_2_ films and fabricated the devices. M.R.J.T. performed the 3D‐printing. Y.L. characterized the material properties and analyzed the data. Y.L., S.D., and W.L. tested the device performance and analyzed the data. Y.D. conducted the in vitro biocompatibility tests. Y.L., M.R.J.T., and S.D established the closed‐loop control circuits. J.A.O., Y.H., Y.Z., A.K., P.H., J.L., and X.C. performed some experiments and provided valuable discussion for this work. Y.L. and P.S.L. wrote the manuscript with the input from all authors. A.I.Y.T., S.J.P., L.E., S.M., and P.S.L. supervised the project. All authors contributed to research and reviewed the manuscript.

## Funding

This research is supported by the National Research Foundation, Prime Minister's Office, Singapore under its Campus of Research Excellence and Technological Enterprise (CREATE) programme, Smart Grippers for Soft Robotics (SGSR).

## Conflicts of Interest

The authors declare no conflicts of interest.

## Supporting information




**Supporting File 1**: advs77323‐sup‐0001‐SuppMat.docx.

**Supporting File 2**: advs77323‐sup‐0002‐S1.mp4.

**Supporting File 3**: advs77323‐sup‐0003‐S2.mp4.

**Supporting File 4**: advs77323‐sup‐0004‐S3.mp4.

**Supporting File 5**: advs77323‐sup‐0005‐S4.mp4.

## Data Availability

All data needed to evaluate the conclusions in the paper are present in the paper and/or the Supplementary Information.

## References

[1] G. Fiori , F. Bonaccorso , G. Iannaccone , et al., “Electronics Based on Two‐Dimensional Materials,” Nature Nanotechnology 9, no. 10 (2014): 768–779, 10.1038/nnano.2014.207. 25286272

[2] C.‐H. Lee , G.‐H. Lee , A. M. Van Der Zande , et al., “Atomically Thin p–n Junctions With Van Der Waals Heterointerfaces,” Nature Nanotechnology 9, no. 9 (2014): 676–681, 10.1038/nnano.2014.150. 25108809

[3] F. Xia , H. Wang , D. Xiao , M. Dubey , and A. Ramasubramaniam , “Two‐Dimensional Material Nanophotonics,” Nature Photonics 8, no. 12 (2014): 899–907, 10.1038/nphoton.2014.271.

[4] W. Choi , N. Choudhary , G. H. Han , J. Park , D. Akinwande , and Y. H. Lee , “Recent Development of Two‐Dimensional Transition Metal Dichalcogenides and Their Applications,” Materials Today 20, no. 3 (2017): 116–130, 10.1016/j.mattod.2016.10.002.

[5] Y. Li , J. A. Ong , and P. S. Lee , “Two‐Dimensional Materials for Adaptive Functionalities in Soft Robotics,” Materials Horizons 12, no. 20 (2025): 8261–8293, 10.1039/D5MH00565E. 40613447

[6] W. Wu , L. Wang , Y. Li , et al., “Piezoelectricity of Single‐Atomic‐Layer MoS_2_ for Energy Conversion and Piezotronics,” Nature 514, no. 7523 (2014): 470–474, 10.1038/nature13792. 25317560

[7] H. Zhu , Y. Wang , J. Xiao , et al., “Observation of Piezoelectricity in Free‐Standing Monolayer MoS_2_ ,” Nature Nanotechnology 10, no. 2 (2015): 151–155, 10.1038/NNANO.2014.309. 25531085

[8] M. N. Blonsky , H. L. Zhuang , A. K. Singh , and R. G. Hennig , “Ab Initio Prediction of Piezoelectricity in Two‐Dimensional Materials,” ACS Nano 9, no. 10 (2015): 9885–9891, 10.1021/acsnano.5b03394. 26312745

[9] Y. Li , R. Goei , A. J. Ong , et al., “Atomic Layer Deposition of Piezoelectric Materials: A Timely Review,” Materials Today Energy 39 (2024): 101457, 10.1016/j.mtener.2023.101457.

[10] S. A. Han , T.‐H. Kim , S. K. Kim , et al., “Point‐Defect‐Passivated MoS_2_ Nanosheet‐Based High Performance Piezoelectric Nanogenerator,” Advanced Materials 30 (2018): 1800342, 10.1002/adma.201800342. 29603416

[11] S. Wang , G. D. Lee , S. Lee , E. Yoon , and J. H. Warner , “Detailed Atomic Reconstruction of Extended Line Defects in Monolayer MoS_2_ ,” ACS Nano 10, no. 5 (2016): 5419–5430, 10.1021/acsnano.6b01673. 27159415

[12] W. Liu , Q. Wang , Y. Zhao , et al., “Siliconizing‐Driven Layer‐by‐Layer Growth of 2D Tellurides With Controlled Crystallization,” Advanced Materials 37 (2025): 2501451, 10.1002/adma.202501451. 40459504

[13] J. Oh , M. Park , Y. Kang , and S.‐Y. Ju , “Real‐Time Observation for MoS_2_ Growth Kinetics and Mechanism Promoted by the Na Droplet,” ACS Nano 18, no. 29 (2024): 19314–19323, 10.1021/acsnano.4c05586. 39001854

[14] C. Liu , X. Xu , L. Qiu , et al., “Kinetic Modulation of Graphene Growth by Fluorine Through Spatially Confined Decomposition of Metal Fluorides,” Nature Chemistry 11, no. 8 (2019): 730–736, 10.1038/s41557-019-0290-1. 31308494

[15] J. Jiang , T. Xu , J. Lu , L. Sun , and Z. Ni , “Defect Engineering in 2D Materials: Precise Manipulation and Improved Functionalities,” Research 2019 (2019): 4641739, 10.34133/2019/4641739. 31912036 PMC6944491

[16] M. Jain , S. Kretschmer , J. Meyer , and A. V. Krasheninnikov , “Adatom‐Mediated Damage of Two‐Dimensional Materials Under the Electron Beam in a Transmission Electron Microscope,” Physical Review Materials 8, no. 5 (2024): 054004, 10.1103/PhysRevMaterials.8.054004.

[17] J.‐G. Song , G. H. Ryu , S. J. Lee , et al., “Controllable Synthesis of Molybdenum Tungsten Disulfide Alloy for Vertically Composition‐Controlled Multilayer,” Nature Communications 6, no. 1 (2015): 7817, 10.1038/ncomms8817. PMC4525162 26204328

[18] R. I. Romanov , M. G. Kozodaev , D. I. Myakota , et al., “Synthesis of Large Area Two‐Dimensional MoS_2_ Films by Sulfurization of Atomic Layer Deposited MoO_3_ Thin Film for Nanoelectronic Applications,” ACS Applied Nano Materials 2, no. 12 (2019): 7521–7531, 10.1021/acsanm.9b01539.

[19] B. D. Keller , A. Bertuch , J. Provine , G. Sundaram , N. Ferralis , and J. C. Grossman , “Process Control of Atomic Layer Deposition Molybdenum Oxide Nucleation and Sulfidation to Large‐Area MoS_2_ Monolayers,” Chemistry of Materials 29, no. 5 (2017): 2024–2032, 10.1021/acs.chemmater.6b03951.

[20] J. V. Pondick , J. M. Woods , J. Xing , Y. Zhou , and J. J. Cha , “Stepwise Sulfurization From MoO_3_ to MoS_2_ via Chemical Vapor Deposition,” ACS Applied Nano Materials 1, no. 10 (2018): 5655–5661, 10.1021/acsanm.8b01266.

[21] S. Fatima , Y. Gu , S. J. Yang , S. Kutagulla , S. Rizwan , and D. Akinwande , “Comparative Study Between Sulfurized MoS_2_ From Molybdenum and Molybdenum Trioxide Precursors for Thin‐Film Device Applications,” ACS Applied Materials & Interfaces 15, no. 12 (2023): 16308–16316, 10.1021/acsami.3c00824. 36939015

[22] Y. Jung , H. Ryu , H. Kim , et al., “Nucleation and Growth of Monolayer MoS_2_ at Multisteps of MoO_2_ Crystals by Sulfurization,” ACS Nano 17, no. 8 (2023): 7865–7871, 10.1021/acsnano.3c01150. 37052379

[23] P. Lin , L. P. Zhu , D. Li , and Z. L. Wang , “Defect Repair for Enhanced Piezo‐Phototronic MoS_2_ Flexible Phototransistors,” Journal of Materials Chemistry C 7, no. 46 (2019): 14731–14738, 10.1039/c9tc05337a.

[24] W. B. Choi , J. Y. Kim , E. H. Lee , G. Mehta , and V. Prasad , “Asymmetric 2D MoS_2_ for Scalable and High‐Performance Piezoelectric Sensors,” ACS Applied Materials & Interfaces 13, no. 11 (2021): 13596–13603, 10.1021/acsami.1c00650. 33710868

[25] S. A. Han , J. Moon , H.‐Y. Yum , M.‐S. Park , S.‐W. Kim , and J. H. Kim , “Suppression of Extra Carriers for Enhanced Intrinsic Piezoelectric Properties of Ultrathin MoS_2_ Through Various Metal Dopants,” Composites Part B: Engineering 246 (2022): 110205, 10.1016/j.compositesb.2022.110205.

[26] A. K. Verma , M. D. A. Rahman , P. Vashishtha , et al., “Oxygen‐Passivated Sulfur Vacancies in Monolayer MoS_2_ for Enhanced Piezoelectricity,” ACS Nano 19, no. 3 (2025): 3478–3489, 10.1021/acsnano.4c13037. 39808476

[27] Y. Han , T. Hu , R. Li , J. Zhou , and J. Dong , “Stabilities and electronic properties of monolayer MoS_2_ With one or two sulfur line vacancy defects,” Physical Chemistry Chemical Physics 17, no. 5 (2015): 3813–3819, 10.1039/c4cp04319g. 25562072

[28] J.‐Y. Lee , J. H. Kim , Y. Jung , et al., “Evolution of Defect Formation During Atomically Precise Desulfurization of Monolayer MoS_2_ ,” Communications Materials 2, no. 1 (2021): 80, 10.1038/s43246-021-00185-4.

[29] Y. Sun , S. Shen , W. Deng , et al., “Suppressing Piezoelectric Screening Effect at Atomic Scale for Enhanced Piezoelectricity,” Nano Energy 105 (2023): 108024, 10.1016/j.nanoen.2022.108024.

[30] Y. S. Jung , H. J. Choi , S. H. Park , et al., “Nanoampere‐Level Piezoelectric Energy Harvesting Performance of Lithography‐Free Centimeter‐Scale MoS_2_ Monolayer Film Generators,” Small 18, no. 24 (2022): 2200184, 10.1002/smll.202200184. 35451217

[31] W. Ma , J. Lu , B. Wan , et al., “Piezoelectricity in Multilayer Black Phosphorus for Piezotronics and Nanogenerators,” Advanced Materials 32, no. 7 (2020): 1905795, 10.1002/adma.201905795. 31930641

[32] F. Xue , J. Zhang , W. Hu , et al., “Multidirection Piezoelectricity in Mono‐ and Multilayered Hexagonal α‐In_2_Se_3_ ,” ACS Nano 12, no. 5 (2018): 4976–4983, 10.1021/acsnano.8b02152. 29694024

[33] Y. Zhang , J. Mao , R.‐K. Zheng , et al., “Ferroelectric Polarization‐Enhanced Performance of Flexible CuInP_2_S_6_ Piezoelectric Nanogenerator for Biomechanical Energy Harvesting and Voice Recognition Applications,” Advanced Functional Materials 33 (2023): 2214745, 10.1002/adfm.202214745.

[34] J.‐H. Lee , J. Y. Park , E. B. Cho , et al., “Reliable Piezoelectricity in Bilayer WSe_2_ for Piezoelectric Nanogenerators,” Advanced Materials 29, no. 29 (2017): 1606667. 10.1002/adma.201606667 28585262

[35] J. Kim , E. Lee , G. Mehta , and W. Choi , “Stable and High‐Performance Piezoelectric Sensor via CVD Grown WS_2_ ,” Nanotechnology 31, no. 44 (2020): 445203, 10.1088/1361-6528/aba659. 32668423

[36] P. Li and Z. Zhang , “Self‐Powered 2D Material‐Based pH Sensor and Photodetector Driven by Monolayer MoSe_2_ Piezoelectric Nanogenerator,” ACS Applied Materials & Interfaces 12, no. 52 (2020): 58132–58139, 10.1021/acsami.0c18028. 33326209

[37] R. Hinchet , U. Khan , C. Falconi , and S. W. Kim , “Piezoelectric Properties in Two‐Dimensional Materials: Simulations and Experiments,” Materials Today 21, no. 6 (2018): 611–630, 10.1016/j.mattod.2018.01.031.

[38] W. Shi , Y. Guo , Z. Zhang , and W. Guo , “Flexoelectricity in Monolayer Transition Metal Dichalcogenides,” The Journal of Physical Chemistry Letters 9, no. 23 (2018): 6841–6846, 10.1021/acs.jpclett.8b03325. 30449097

[39] C. M. Ganski , A. C. De Palma , and E. T. Yu , “Enhanced Electromechanical Response Due to Inhomogeneous Strain in Monolayer MoS_2_ ,” Nano Letters 24, no. 26 (2024): 7903–7910, 10.1021/acs.nanolett.4c01126. 38899791

[40] Z. Zhang , S. Liu , Q. Pan , et al., “Van der Waals Exfoliation Processed Biopiezoelectric Submucosa Ultrathin Films,” Advanced Materials 34, no. 26 (2022): 2200864, 10.1002/adma.202200864. 35470922

[41] D. K. Patel , A. H. Sakhaei , M. Layani , B. Zhang , Q. Ge , and S. Magdassi , “Highly Stretchable and UV Curable Elastomers for Digital Light Processing Based 3D Printing,” Advanced Materials 29, no. 15 (2017): 1606000, 10.1002/adma.201606000. 28169466

