# Supplementary material for: Monolayer MoS2 With Ultrahigh Piezoelectricity: From ALD Dose Control to Device Performance
Source: Adv Sci (Weinh). 2026 Aug 21:e77323. Online ahead of print. doi: 10.1002/advs.77323 (PMC13496285; doi:10.1002/advs.77323)
Supplement: Supplementary file 1 [file ADVS-9999-e77323-s003.docx]

Supporting Information

**Monolayer MoS_2_ with Ultrahigh Piezoelectricity: From ALD Dose Control to Device Performance**

*Yun Li, Ming Rui Joel Tan, Subhasis Das, Yafei Du, Wenjie Lai, Jiamin Amanda Ong, Yongli He, Yiming Zou, Anatoli Kurkin, Peiwen Huang, Jiajun Liu, Xingyu Chen, Alfred Iing Yoong Tok, Soo Jay Phee, Lioz Etgar, Shlomo Magdassi*, and Pooi See Lee**

**Table S1:** Deposition recipes for different growth paths.

| **Mode** | **Pulse of Mo(CO)_6_** | **Holding of Mo(CO)_6_** | **Repetition sub-cycles of Mo(CO)_6_** | **Pulse of O_3_** | **Holding of O_3_** | **Purge** |
| --- | --- | --- | --- | --- | --- | --- |
| **Path 1** | 400 ms | N/A | 1 | 2000 ms | N/A | 30 s |
| **Path 2** | 400 ms | 15 s | 1 | 2000 ms | 8 s | 30 s |
| **Path 3** | 400 ms | 15 s | 3 | 2000 ms | 8 s | 30 s |

**Table S2:** Parameters to deposit P-ML and C-ML.

| **Sample** | **Pulse-holding repetition times of Mo(CO)_6_** | **Number of cycles** |
| --- | --- | --- |
| **P-ML** | 1 | 12 |
| **C-ML** | 3 | 5 |

**Table S3:** Relative fractions of Mo^6+^, Mo^4+^, and MoO_x_S_y_ components obtained from peak fitting of the intermediate Mo 3d XPS spectra of P-ML and C-ML sulfurized at 600 °C for 10 min.

|  | **P-ML** | **C-ML** |
| --- | --- | --- |
| Mo^4+^ | 0.37 | 0.75 |
| Mo^6+^ | 0.40 | 0.05 |
| MoO_x_S_y_ | 0.23 | 0.20 |

**Table S4:** Summary of the state-of-art piezoelectric sensors based on 2D materials.

| **Materials** | **Device Type** | **Area/μm^2^** | **Sensitivity, per 1% strain** | **Detection limit** | **Ref.** |
| --- | --- | --- | --- | --- | --- |
| Mechanically exfoliated **MoS_2_**, single-layer | Cantilever bending | 25 | 55.1 pA and 32.8 mV, linear | Ꜫ=0.21%, 0.5Hz | ^1^ |
| Mechanically exfoliated **Te-doped BP**, multi-layer | Convex bending | 50 | ~6pA, non-linear | Ꜫ=0.16% | ^2^ |
| Mechanically exfoliated **α-In_2_Se_3_**, multi-layer | Cantilever bending | 5 000 | ~50.31 pA, non-linear | Ꜫ=0.18% | ^3^ |
| CVD **MoS_2_**, single-layer with S vacancy passivation | Convex bending | 10^4^ | ~65.38 mV and 539 pA (fixed velocity with 60mm/s, changing Ꜫ and frequency simultaneously) | Ꜫ=0.28%, 5.9 Hz (60mm/s) | ^4^ |
| CVT **CuInP_2_S_6_**, multi-layer with single crystal | Convex bending | 40 | ~80 mV, linear from 0.65%-0.91% strain | N/A | ^5^ |
| CVD **WSe_2_**, bilayer | Convex bending | 5 000 | ~208 mV, non-linear | ~Ꜫ=0.28%, 0.3 Hz (40mm/s) | ^6^ |
| CVD **MoS_2_**, single layer with preferred orientation | Convex bending | 18.3 × 10^6^ | N/A, peak output is 400.4mV and 40.7nA | Ꜫ=0.57%, 2Hz (100 mm/s) | ^7^ |
| CVD **WS_2_**, single layer with solvent-vapor annealing | Tapping | N/A | 0.756 pA kPa^-1^  19.8 mV kPa^-1^ | 1 kPa | ^8^ |
| CVD **MoSe_2_**, single layer | Convex bending | N/A | 100 mV | Ꜫ=0.2% | ^9^ |
| CVD **MoS_2_**, with Oxygen-Passivated Sulfur Vacancies | Dynamic compression | 1000 | 0.05 V, non-linear | 0.2 mm amplitude, 5Hz | ^10^ |
| C-ML | Convex bending | 10.5 × 10^6^ | 326.9 mV, 141.38 pA, linear | Ꜫ= 0.2%, 0.5Hz (2.5 mm/s) | **This work** |

**Figure S1:** Schematic diagram of the fabrication process of ultrathin MoS_2_ piezoelectric device. MoO_x_ film is deposited via ALD, followed by sulfurization to MoS_2_ in tube furnace. The resulting film is transferred onto PET substrate with patterned side electrodes to integrate a piezoelectric device.

**Figure S2:** The method to control ALD dose through the precursor pulse pressure and exposure duration.

**Figure S3:** Peak-fitted Mo 3d and S 2p XPS spectra of the stepwise sulfurization intermediates for P-ML and C-ML. Spectra were collected after sulfurization at 600 °C for 5 min and 10 min, showing distinct intermediate sulfurization states for the two samples under identical treatment conditions.

**Figure S4:** AFM images of different samples with distinct coverage of monolayers by tuning the growth temperature (155 °C-165 °C) and ALD dose. ALD recipe: (400ms Pulse of Mo(CO)_6_ - 15s holding of precursor - 30s purge) repeating n times - 2000 ms pulse of O_3_ - 8s holding of O_3_ - 30s purge, where n = 1, 2, and 3 for ALD dose 1, 2, and 3.

**Figure S5:** Coverage percentage of monolayers in Figure S4.

**Figure S6:** Water contact angle (WCA) comparison between different surface treatments. a, WCA on sapphire substrates before and after 30 mins at 165 ^o^C with different treatments. b, WCA images on sapphire after 30 mins at 165 ^o^C with different treatments. Plasma treatment (10 mins, 300 W, Ar and O_2_) was selected as the pre-treatment for substrates to ensure the effective adsorption of precursors.

**Figure S7:** Mechanisms for temperature-induced decomposition, adsorption, and possible diffusion of precursor Mo(CO)_6_. ALD window is 155-165 ^o^C (a) but the growth rate is increasing with the temperatures within this window (b). This is due to the partial decomposition of precursors (c, d) under these temperatures, allowing smaller volume of adsorbed species, more adatoms, and promoted diffusion with higher temperatures.

**Figure S8:** AFM images of MoS_2_ films after 5 cycles by Path 3 at different temperatures. a, 150 ^o^C. b, 169 ^o^C. c, 173 ^o^C. This indicates that the deposition outside the optimal temperature window cannot provide high quality film due to the uncontrolled thermodynamic process.

**Figure S9:** TEM images of polycrystal P-ML and C-ML. Top-view TEM images with corresponding FFT images of P-ML(a,c) and C-ML (b,d).

**Figure S10:** Raman mapping of P-ML and C-ML.

**Figure S11:** XPS spectra in O 1s and C 1s of P-ML and C-ML.

**Figure S12:** Film quality after wet-transfer process. a, Optical image of transferred MoS_2_ on SiO_2_/Si substrate. b, c, Optical microscopic images of transferred MoS_2_. d, AFM image of transferred MoS_2_.

**Figure S13:** Strain calculation in device measurements. a. bending strain calculation. b, Stretching strain calculation.

**Figure S14:** Output voltage of C-ML under different bending strain with bending frequency of 0.5Hz. a, 0.37% strain. b, 0.52% strain. c, 0.72% strain. d, sensitivity.

**Figure S15:** Raman and PL of monolayer MoS_2_ as a function of the uniaxial strain by bending and stretching. a, b, Stretching. c, d, Bending.

**Figure S16:** a, Schematic of the competing effect of defects on piezoelectricity. Defect-induced strain and asymmetry locally enhance piezoelectricity, while screening effects decrease piezoelectricity. b, Finite element analysis (FEA) of strain distribution in monolayer MoS_2_ under bending: left, continuous film; right, film with a pinhole. c, The C-ML/P-ML piezoelectric ratio drops from ~5 at the microscale to ~2.3 at the macroscale.

**Figure S17:** Representative images of live/dead staining (Green and red labels indicate living and dead cells, respectively) and DAPI/ phalloidine staining (red and blue labeled cells represent cytoskeleton and nucleus of cells, respectively) of HEK 293T and BBM cells after 3 days culturing with and without MoS_2_ films.

**Figure S18:** Illustration of the bio-inspired slippage closed-loop control for smart grippers. Inspired by human gripping and slippage feedback, artificial slippage feedback is achieved in a pneumatic soft gripper, where MoS_2_-based flexible slippage sensor (FSS) acts as slippage receptor to provide real-time slippage signal to decision maker, followed by adaptive gripping force regulation.

**Figure S19:** FSS and pressure sensor signals and photos during a sole slippage detection event without closed-loop control, which failed to pick up the wax apple.

**Figure S20:** Compatibility with this closed-loop feedback for picking various objects. a, boxed snack (hard and flat). b, bell pepper (big and smooth). c, cake (small and soft).

**Figure S21:** Adaptive pressure feedback achieved by the closed-loop control system, using boxed snack as an example. a, successful lift with closed-loop control and initial 30 kPa. b, fail lift without closed-loop control and initial 35 kPa. c, the minimum static lifting pressure is ~38 kPa, successful lift without closed-loop control and initial 38 kPa. This indicates that our system is adaptive feedback control.

**Figure S22:** Design of fully 3D-printing pneumatic finger.

**Note S1 Two-step growth design and rationale for isolating the effect of ALD dose**

A two-step process was used in this work to prepare monolayer MoS_2_. In Step 1, an ultrathin MoO_x_ film was deposited by ALD, which defined the initial oxide-film state and the eventual thickness of the sulfurized product. In Step 2, the as-deposited MoO_x_ film was converted into MoS_2_ by sulfurization at elevated temperature to obtain highly crystalline films.

To examine how the ALD step governs the final film outcome, the sulfurization recipe was kept identical for all samples throughout this work. Under this controlled two-tep scheme, differences in the final MoS_2_ films are therefore attributed primarily to variations established during the ALD deposition of the precursor oxide film, rather than to differences in the conversion conditions. This design is consistent with the growth overview in Figure 1, where the as-deposited MoO_x_ film state governs the subsequent sulfurization pathway and the final MoS_2_ morphology.

Prior to ALD, sapphire substrates were treated by oxygen plasma to increase surface hydrophilicity and promote precursor adsorption (See **Figure S6**). The ALD temperature was fixed at 165 °C for the compared growth routes, because this condition was identified as the optimized deposition window (See **Figures S7 and S8**) for achieving effective adsorption and controlled oxide-film formation. At this temperature, partial decomposition of Mo(CO)_6_ facilitates adsorption and helps shorten the ALD incubation period, thereby improving ultrathin-film formation.

During the ALD step, precursor holding was introduced to improve adsorption uniformity within each cycle and to achieve better thickness control with less precursor waste. The effective precursor dose was adjusted through the dosing scheme illustrated in Figure S2 by varying the number of precursor pulse-hold-purge sub-cycles in each ALD cycle, while the oxidant step was kept unchanged. The detailed deposition recipes for the different growth pathways are summarized in **Tables S1and S2.**

Because the sulfurization atmosphere was sulfur-rich and identical for all samples, additional variability arising from sulfur supply during conversion was minimized. Likewise, keeping the ALD temperature constant excluded temperature-dependent thermodynamic effects across the compared routes. Therefore, the effective precursor dose during the ALD step is the primary experimental variable controlling the quality of the as-deposited MoO_x_ film and, consequently, the morphology and defect characteristics of the resulting MoS_2_ films.

**Note S2 Characterization of ALD MoO_x_ films in Path 2 and Path 3**

To further support the proposed growth picture and clarify the role of the oxide film, we performed comparative characterization of the as-deposited MoO_x_ films obtained from Path 2 and Path 3 before sulfurization. These two pathways were selected because both yield monolayer MoS_2_ after sulfurization, allowing the effect of the ALD dose on oxide-film quality to be examined without the added influence of large thickness differences associated with the other growth pathways.

AFM topography images and corresponding 3D surface plots reveal distinct surface roughness characteristics of the MoO_x_ films deposited in Path 2 and Path 3. The Path 2 MoO_x_ film exhibits a relatively rougher surface with an average roughness (Ra) of 0.36 nm, characterized by more pronounced nanoscale features. In contrast, the Path 3 MoO_x_ film shows a smoother morphology with a lower roughness value of 0.12 nm, indicating fewer surface irregularities (**Figure S23**). These results indicate that increasing the effective precursor dose during ALD improves the uniformity of the as-deposited oxide film.

**Figure S23:** Surface morphological comparison of MoO_x_ films in Path 2 and Path 3 (both converted to MoS_2_ monolayers after sulfurization).

We further compared the thicknesses of the two oxide films by AFM (Figure S24). Multiple height profiles collected from each sample show that the average thicknesses are 1.082 $\pm$0.016 nm for Path 2 and 1.064 $\pm$0.028 nm for Path 3, demonstrating that the two oxide films have comparable thickness prior to sulfurization, as shown in Figure S24. This comparison indicates that the distinct sulfurization outcomes and final MoS_2_ morphologies do not originate primarily from differences in oxide-film thickness.

**Figure S24:** Thickness comparison of MoO_x_ films in Path 2 and Path 3 (both converted to MoS_2_ monolayers after sulfurization).

High-resolution XPS analysis further reveals differences in the chemical state of the precursor oxide films (Figure S25). In both samples, the Mo 3d and O 1s spectra are dominated by Mo in the Mo^6+^ oxidation state, consistent with ultrathin MoO_x_ precursor layers. However, the Mo 3d peaks of the Path 3 film shift slightly toward higher binding energies, to 233.37 eV and 236.47 eV, compared with 233.25 eV and 236.35 eV for Path 2, as shown in Figure S25. The corresponding metal-oxygen O 1s peak also shifts higher by about 0.15 eV in Path 3, from 531.05 eV to 531.20 eV. In addition, the O/Mo atomic ratio derived from the metal-oxygen component is higher for Path 3 than for Path 2, with values of 3.60 and 3.45, respectively. Although the absolute values exceed the stoichiometric value because of the ultrathin nature of the films and substrate contributions, the relative difference still indicates that the Path 3 oxide film is more stoichiometric and contains fewer oxygen-deficient features.

**Figure S25:** XPS spectra in Mo 3d and O 1s of MoO_x_ films in Path 2 and Path 3 (both converted to MoS_2_ monolayers after sulfurization).

**Note S3 Lateral piezoresponse force microscopy measurements and calibration**

Lateral piezoresponse force microscopy (LPFM) was employed to probe the in-plane electromechanical response of the MoS_2_ samples. All measurements were carried out on a Bruker Dimension Icon atomic force microscope using a Pt/Ir-coated conductive probe (SCM-PIT, Bruker) with a nominal spring constant of 3 N m^-1^ and a nominal resonance frequency of 75 kHz. In the present measurement geometry, the AC excitation voltage was applied through the laterally patterned Au electrodes to generate an in-plane electric field across the MoS_2_ channel.

As illustrated in Figure S26a, the in-plane deformation induced by the lateral electric field leads to a torsional motion of the cantilever, which is detected by the position-sensitive photodetector through the lateral displacement of the reflected laser beam. Figure S26b shows an optical micrograph of the actual measurement configuration, in which the probe was positioned within the electrode gap and brought into contact with the selected region of the flake. Care was taken to maintain stable tip-sample contact and to position the probe away from obvious electrode-edge artifacts during the measurements. Because the measured LPFM signal may include contributions from local contact mechanics, electrostatic coupling, and geometry-dependent cantilever transduction, the extracted coefficient is reported throughout this work as an effective in-plane piezoelectric coefficient, $d_{11}^{\mathrm{eff}}$, rather than the intrinsic tensor element $d_{11}$.

Before the quantitative measurements, the frequency response of the tip-sample junction was examined to identify the contact resonance behavior. As shown in Figure S26c, a clear resonance peak was observed at approximately 336 kHz, confirming stable contact between the probe and the sample. To reduce resonance-enhanced artifacts and to maintain a stable electromechanical readout, the AC drive frequency used for the quantitative LPFM measurements was selected well below the contact resonance frequency (15 kHz). This sub-resonant operation helps suppress excessive resonance amplification and improves the reliability of the quantitative analysis.

For quantitative analysis, the raw LPFM amplitude recorded by the lock-in amplifier, which is initially expressed in voltage units, was converted into real displacement using the calibrated PFM sensitivity. The normal deflection sensitivity of the optical beam deflection system was first determined from the slope of the force-distance curve acquired in contact mode on a hard and flat sapphire substrate, as shown in Figure S26d. From this calibration, an out-of-plane deflection sensitivity $S_{\mathrm{OOP}}$of approximately 112 nm V^-1^ was obtained. Since LPFM detects cantilever torsion associated with in-plane shear deformation, the in-plane sensitivity cannot be directly taken from the force-curve calibration. Instead, the in-plane sensitivity $S_{\mathrm{IP}}$ was derived from the calibrated out-of-plane sensitivity and the cantilever geometry according to $S_{\mathrm{OOP}}=S_{\mathrm{IP}}\times2L/3h$, where $L$is the cantilever length and $h$is the tip height.^11^ For the SCM-PIT probe, the nominal values $L=225 \mu m$and $h=15 \mu m$give an out-of-plane to in-plane sensitivity ratio of 10, yielding $S_{\mathrm{IP}}\approx11.20$nm V^-1^.

In addition, a background LPFM signal was acquired under the same measurement conditions on the sapphire substrate or on a non-piezoelectrically active region and was used to evaluate the instrumental and non-piezoelectric contribution to the measured response. Because voltage-modulated AFM measurements can contain undesired background contributions, including electrostatic interactions and contact-related artifacts, the background signal was subtracted from the measured LPFM amplitude prior to displacement conversion and linear fitting. The background-corrected LPFM amplitude was then converted into in-plane displacement using the calibrated $S_{\mathrm{IP}}$, and quantitative single-point measurements were performed by varying the AC voltage applied across the lateral electrodes under fixed contact conditions. The effective in-plane piezoelectric coefficient, $d_{11}^{\mathrm{eff}}$, was extracted from the linear dependence of the background-corrected displacement amplitude on the applied AC voltage.

**Figure S26.** LPFM measurement setup and calibration. a, Schematic of the lateral electrode-driven LPFM configuration. b, Optical image of the probe position in the electrode gap. c, Contact resonance spectrum. d, Force-distance curve on sapphire for sensitivity calibration and background assessment.

**Figure S27.** Figure S27. Large-area Kelvin probe surface potential maps of (a) P-ML and (b) C-ML MoS_2_ films. The higher surface potential of C-ML is consistent with the KPFM results in Figure 2i, confirming the same surface-potential trend observed by KPFM over a larger area.

**References**

1	Wu, W. Z. *et al.* Piezoelectricity of single-atomic-layer MoS_2_ for energy conversion and piezotronics. *Nature* **514**, 470, doi:10.1038/nature13792 (2014).

2	Ma, W. D. *et al.* Piezoelectricity in Multilayer Black Phosphorus for Piezotronics and Nanogenerators. *Adv. Mater.* **32**, doi:10.1002/adma.201905795 (2020).

3	Xue, F. *et al.* Multidirection Piezoelectricity in Mono- and Multilayered Hexagonal alpha-In_2_Se_3_. *ACS Nano* **12**, 4976-4983, doi:10.1021/acsnano.8b02152 (2018).

4	Han, S. A. *et al.* Point-Defect-Passivated MoS_2_ Nanosheet-Based High Performance Piezoelectric Nanogenerator. *Adv. Mater.* **30**, doi:10.1002/adma.201800342 (2018).

5	Zhang, Y. *et al.* Ferroelectric Polarization‐Enhanced Performance of Flexible CuInP_2_S_6_ Piezoelectric Nanogenerator for Biomechanical Energy Harvesting and Voice Recognition Applications. *Adv. Funct. Mater.* **33**, doi:10.1002/adfm.202214745 (2023).

6	Lee, J. H. *et al.* Reliable Piezoelectricity in Bilayer WSe_2_ for Piezoelectric Nanogenerators. *Adv. Mater.* **29**, doi:10.1002/adma.201606667 (2017).

7	Jung, Y. S. *et al.* Nanoampere-Level Piezoelectric Energy Harvesting Performance of Lithography-Free Centimeter-Scale MoS_2_ Monolayer Film Generators. *Small*, doi:10.1002/smll.202200184.

8	Kim, J., Lee, E., Mehta, G. & Choi, W. Stable and high-performance piezoelectric sensor via CVD grown WS_2_. *Nanotechnology* **31**, 445203, doi:10.1088/1361-6528/aba659 (2020).

9	Li, P. & Zhang, Z. Self-Powered 2D Material-Based pH Sensor and Photodetector Driven by Monolayer MoSe_2_ Piezoelectric Nanogenerator. *ACS Appl. Mater. Interfaces* **12**, 58132-58139, doi:10.1021/acsami.0c18028 (2020).

10	Verma, A. K. *et al.* Oxygen-Passivated Sulfur Vacancies in Monolayer MoS_2_ for Enhanced Piezoelectricity. *ACS Nano* **19**, 3478-3489, doi:10.1021/acsnano.4c13037 (2025).

11	Zhang, Z. *et al.* Van der Waals Exfoliation Processed Biopiezoelectric Submucosa Ultrathin Films. *Adv. Mater.* **34**, 2200864, doi:<https://doi.org/10.1002/adma.202200864> (2022).
